# Direct and indirect influences of intercrops on the coconut defoliator *Opisina arenosella*

**DOI:** 10.1007/s10340-017-0904-6

**Published:** 2017-08-09

**Authors:** K. S. Shameer, M. Nasser, Chandrika Mohan, Ian C. W. Hardy

**Affiliations:** 1 Insect Ecology and Ethology Laboratory, Department of Zoology, University of Calicut, Calicut University P.O., Malappuram, Kerala 673635 India; 2 Department of Entomology, ICAR - Central Plantation Crops Research Institute, Regional Station, Kayamkulam, Kerala 690533 India; 3 School of Biosciences, University of Nottingham, Sutton Bonington Campus, Loughborough, Leicestershire, LE12 5RD UK

**Keywords:** Plant–herbivore–parasitoid associations, Trophic connectance webs, Apparent competition, Natural enemy maintenance, Coconut intercrops

## Abstract

Coconut palm (*Cocos nucifera*) infestation by *Opisina arenosella* (Lepidoptera: Oecophoridae) in the Indian subcontinent may occur in November to May each year in the same or adjoining areas of plantations. Parasitoids of *O. arenosella* may also be consistently present at these times. During other periods, pests and/or parasitoids could be maintained on intercrops that are commonly grown throughout the year. Field surveys of 54 intercrop species in Kerala, India, found that *O. arenosella* attacks banana, but not others, while laboratory screening showed that *O. arenosella* can mature on jack fruit, cashew and oil palm. Larvae of 20 lepidopteran species found on intercrops were screened for use by *Goniozus nephantidis* (Hymenoptera: Bethylidae), a larval parasitoid of *O. arenosella*, which oviposited on two species but its offspring failed to mature. Thirteen intercrop herbivore species were screened for use by *Brachymeria nosatoi* (Hymenoptera: Chalcididae), a pupal parasitoid of *O. arenosella*, which completed development on the pyralids *Herculia nigrivita*, *Syllepte derogata* and *Psara basalis*. Further, connectance trophic webs were compiled using prior field records of coconut, 33 species of intercrops, 58 species of lepidopteran herbivores and 29 species of primary parasitoids. Both laboratory and literature evidence suggests that populations of *O. arenosella* are unlikely to be maintained by feeding on intercrops or strongly influenced by direct competition with other lepidopterans but are likely to be affected by sharing parasitoids. Intercrop herbivores have clear potential for maintaining parasitoids of *O. arenosella,* and we recommend thirteen plant species as intercrops that should aid in conservation biocontrol.

## Key message

- Intercrop plants may harbour pests and their natural enemies. The pros and cons of intercropping are likely to vary across agro-ecosystems.
- In coconut plantations, intercrops are little utilized by the major pest of coconut, nor do intercrop herbivores substantially attack coconut. Direct ecological interactions are thus likely to be weak.
- There is a considerable degree of shared parasitism between coconut and intercrop herbivores. Pest populations could thus be suppressed by indirect interactions. Intercrop species are recommended to promote such effects.

## Introduction

**Fig. 1:**
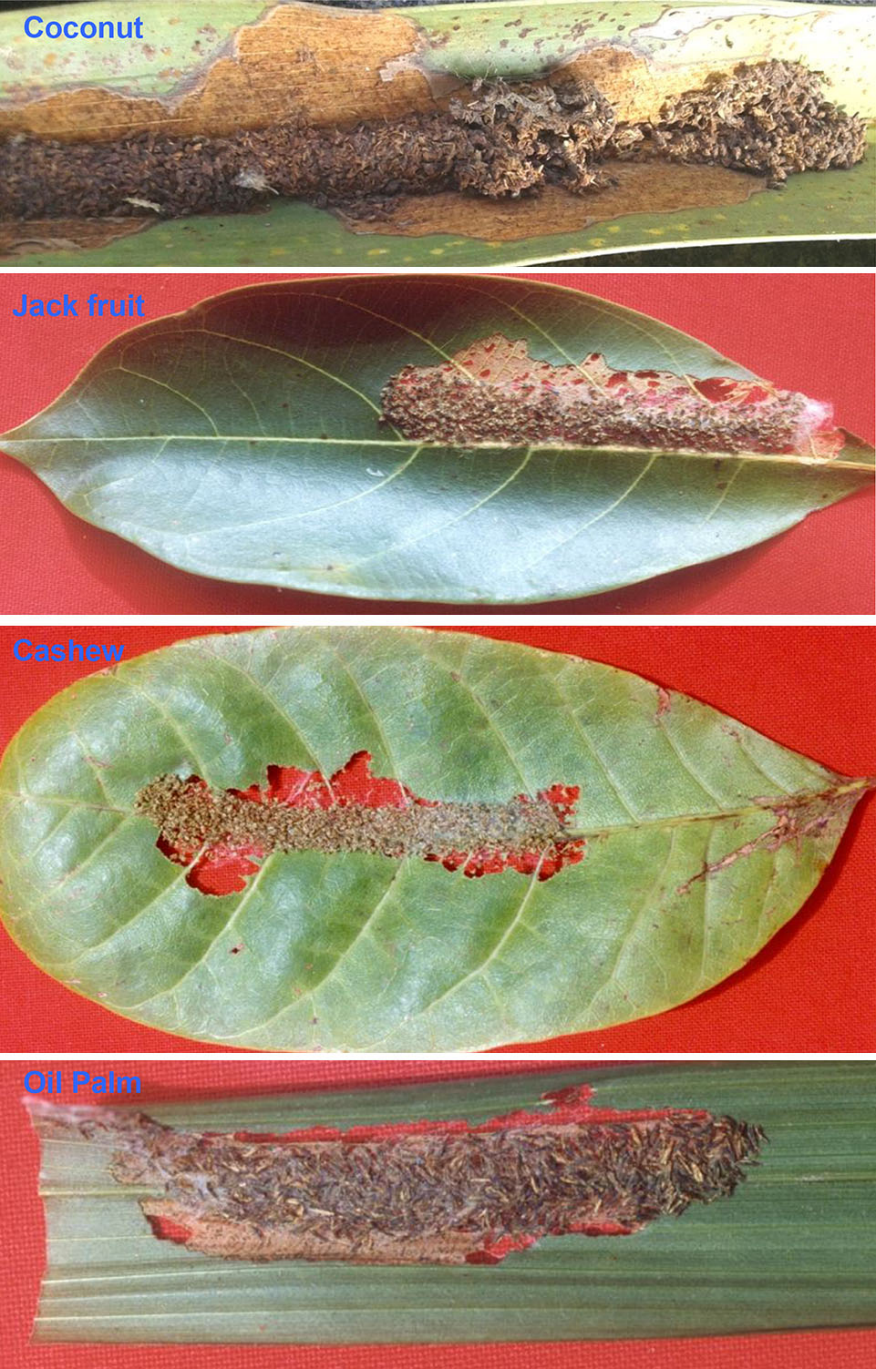
Characteristic feeding galleries made by individual *O. arenosella* larvae on coconut (*upper panel*) and on the leaves of different intercrop plants

The coconut palm, *Cocos nucifera* L., is grown in more than 93 countries, in areas totalling 12,479 million Ha, and yields harvest in all seasons. It is regarded in tropical countries as the ‘Tree of Life’ (Foale 2003). In India, the coconut cultivation industry directly or indirectly employs approximately 12 million people and contributes 1.28 billion USD to GDP (Thomas 2013). Coconut is, however, attacked by more than 800 species of pests. In India and Sri Lanka, one of the major pests is the coconut leaf eating caterpillar *Opisina arenosella* Walker (Lepidoptera: Oecophoridae), with outbreaks causing serious damage to coconut and other palms, typically via feeding on the underside of leaves whilst protected by a gallery made of frass and silken threads (Nirula 1956; Mohan and Sujatha 2006; Singh and Rethinam 2006; Kumara et al. 2015; Fig. 1.). For instance, Mohan et al. (2010) reported that the nut yield of infested coconut palms could be reduced by as much as 45.4% in the year following severe pest incidence and also that the number of flower bunches and leaves could be reduced by 21 and 13.8%, respectively. *Opisina arenosella* is also reported to infest a number of other species of palms (palmyra palm, *Borassus flabellifer* Linn., Rao et al. 1948; Murthy et al. 1995; date palm, *Phoenix dactylifera* Linn., Butani 1975; Talati and Kapadia 1984; fan palm, *Livistona chinensis*, wild date palm, *Phoenix sylvestris*, Talati and Kapadia 1984; talipot palm, *Corypha umbraculifera* Linn., Talati and Kapadia 1984; Sadakathulla et al. 1999).

*Opisina arenosella* occurrence varies seasonally, with both high temperatures and high humidity reported to favour the build-up of populations on coconut palms (Sathiamma et al. 1973; Narendran et al. 1978; Nadarajan and Channabasavanna 1980). In south India, oviposition is most common from November to March and the highest abundance of early instar larvae is observed during November to December (Nadarajan and Channabasavanna 1980) but overall the largest numbers of *O. arenosella* may be observed between February and May (Narendran et al. 1978).

There is relatively little known about the abundance and activity of *O. arenosella* during the period in which coconut palm is not typically infested (June to October). One possibility is that the pest population is maintained by utilizing non-palm plant species. In other cropping systems, alternative host plants can support pests during periods when primary hosts are seasonally unavailable, and subsequently these pests migrate back to the primary host plants (Clementine et al. 2005; Goodell 2009; Saeed et al. 2015) and the availability, density and type of alternative host plants can be important factors influencing the damage caused by insect pests (Power 1987; Settle et al. 1996; Atakan and Uygur 2005; van Veen et al. 2006a; Zhang et al. 2017; but see Feng et al. 2017). Such alternative host plants are potentially present in the coconut agro-ecosystem because a wide variety of intercrop species are commonly grown within plantations and at all times of the year. The height of the coconut palms and the orientation of leaves allow 20–50% of sunlight transmission to reach the ground, making it possible for many annual and perennial plants to be grown in the spaces between coconut trunks (Nelliat et al. 1974), ideally without incurring substantial yield loss in the main crop (Letourneau et al. 2011; Iverson et al. 2014). The common intercrops recommended to be grown with coconut include banana, cocoa, pineapple, tuber crops (tapioca, colocasia, yam), spices (clove, black pepper, nutmeg, ginger) and vegetables (Balasundaram and Aiyadurai 1963; Varghese et al. 1978; Bavappa et al. 1986; Hegde et al. 1993). It is already known that *O. arenosella* is able to infest banana plants (*Musa paradisiaca* L.) (Talati and Butani 1988; Manjunath 1985), but there is little information on whether the remaining intercrop plants can act as alternative hosts and thus support populations of this pest.

*Opisina arenosella* is attacked by a number of species of indigenous natural enemies, including parasitoids. The early larval parasitoid, *Apanteles taragammae* Viereck (Hymenoptera: Braconidae), the late-larval parasitoid *Goniozus nephantidis* (Muesebeck) (Hymenoptera: Bethylidae), the pre-pupal parasitoid *Elasmus nephantidis* Rohwer (Hymenoptera: Elasmidae) and the pupal parasitoid *Brachymeria nosatoi* Habu (Hymenoptera: Chalcididae) are all typically found in coconut plantations in Kerala during the *O. arenosella* infestation period, i.e. November to May, each year (S.K.S. pers. obs.). These are considered to be the most important natural enemies of *O. arenosella.* The release of *G. nephantidis*, *E. nephantidis* and *B. nosatoi* at fixed rates and intervals can result in a significant pest population reduction (Sathiamma et al. 1987, 1996). Estimates of parasitism rates range from 4.23 to 59.50% for *A. taragamae*, 19.57% for *G. nephantidis* and 41.6% for *Brachymeria* spp. (chiefly *B. nosatoi*) (Mohamed et al. 1982; Mohan and Sujatha 2006). Although many predators of *O. arenosella*, such as mites, ants, spiders, anthocorid bugs, are reported, none of these exhibit host attack rates or achieve the same population suppression as the parasitoids.

As with *O. arenosella*, there is relatively little known about the activity of natural enemies during June to October but it is possible that parasitoid populations are maintained by reproduction on pests that infest the intercrop plant species in coconut plantations. To date, the laboratory evaluations of the suitability of different lepidopteran species as hosts for mass-rearing parasitoids of *O. arenosella* have shown that both *G. nephantidis* and *B. nosatoi* can be reared on some alternative host species (Dharmaraju 1952; Mohamed et al. 1982, 1983; Remadevi et al. 1996; Shameer and Mohan 2002; Mohan and Shameer 2003). As with other cropping systems (Settle et al. 1996; Valladares and Salvo 1999; Goodell 2009; Koji et al. 2012; Saeed et al. 2015), reproduction of natural enemies on alternative hosts, themselves feeding on alternative host plants, could influence the suppression of *O. arenosella* damage to coconut production.

Here, we evaluate the possibility that *O. arenosella* populations utilize intercrop plants and, similarly, the possibility that populations of its natural enemies also attack alternative hosts which are found on these intercrop plants. We do this by directly surveying intercrop plants present within the coconut cropping system and testing the ability of *O. arenosella* to feed and develop on these plant species. We also test the ability of some common parasitoids of *O. arenosella* to develop on lepidopteran herbivores of intercrops. Additionally, we construct, from prior literature, connectance trophic webs (Memmott and Godfray 1994) of the coconut agro-ecosystem: such trophic networks record the presence and absence of trophic interactions between coconut and intercrop plants and their herbivores and between these herbivores and their parasitoids. We use these heuristically to further infer the host-plant range of the herbivores, the host range of the parasitoids and the importance of direct (e.g. competition) and indirect (e.g. apparent competition) interactions on *O. arenosella* populations. We conclude by recommending intercrop species to be grown within the coconut agro-ecosystem to promote the conservation biocontrol of *O. arenosella*.

## Materials and methods

### Field survey of intercrop plants

**Fig. 2:**
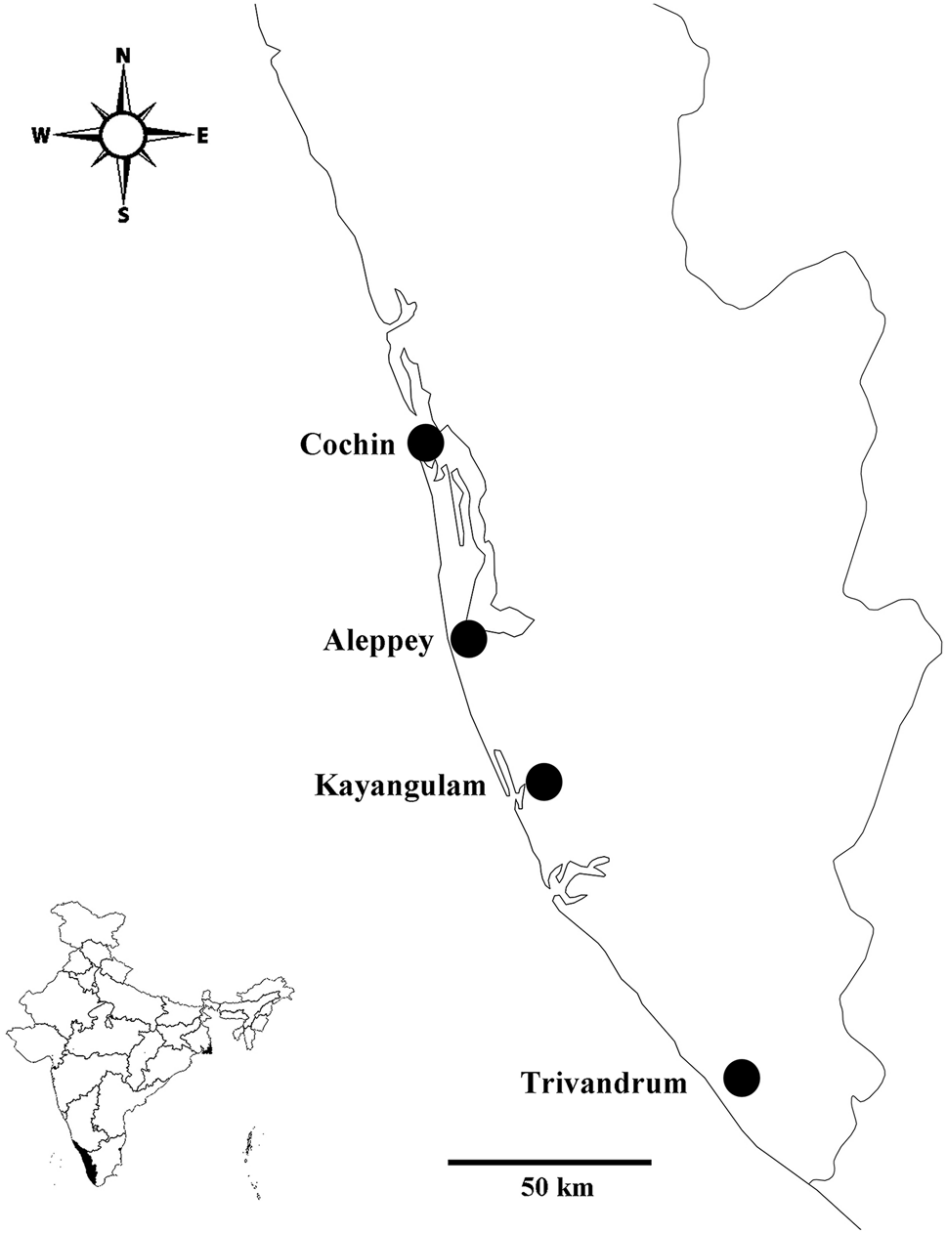
Locations of field sites within Kerala Cochin (Kochi): 09°57′N Latitude, 76°16′E Longitude. Aleppey (Alappuzha): 9°5′N Latitude, 76°33′E Longitude. Kayangulam (Kayamkulam): 9°8′N Latitude, 76°30′E Longitude. Trivandrum (Thiruvananthapuram) (08°29′N Latitude, 76°57′E Longitude). *Inset*: Map of India showing Kerala in the south west. (Map constructed using SimpleMappr, Shorthouse 2010)

Field observations were conducted in four geographically similar locations within Kerala twice each year, during the pest infestation (November to May) and non-infestation periods (June to October), for 2 years (2010–11 and 2011–12). These locations were spread approximately evenly over a ~200 km distance, from north to south: Cochin, Aleppey, Kayangulam and Trivandrum (Fig. 2). The maximum temperature of these locations was 32–34 °C (means for each location were 29, 29, 28 and 27 °C, respectively) and the average relative humidity was 84–90%, with annual rainfall of 1700–2700 mm (Meteorological Centre, Trivandrum, http://www.imdtvm.gov.in/).

In each period and at each location, all the available intercrops grown in coconut plantations and other commonly cultivated crops, including ornamental plants, in coconut plantations and nearby areas (those immediately abutting the plantations, typically fields of rice) were surveyed. A minimum of five coconut plantations in which intercrops were abundant were surveyed in each location. In total, 54 intercrop species were found and at least 50 plants of each species were observed (Table 1). The incidence of *O. arenosella* on these plant species and also the presence of other species of lepidopteran larvae were recorded.

**Table 1:** Intercrop plants surveyed in the field*

Family	Species	Common name	Host type	Type of plant
Amaranthaceae	*Amaranthus viridis* L.	Slender amaranth	Vegetable	Herb
Anacardiaceae	*Anacardium occidentale* L.	Cashew apple, Cashew-nut tree	Crop	Tree
	*Mangifera indica* L.	Mango tree	Fruit	Tree
Annonaceae Araceae	*Annona squamosa* L.	Custard apple	Fruit	Tree
	*Amorphophalus paeonifolius* (Dennst.) Nicolson	Elephant foot yam	Vegetable/tuber crop	Herb
	*Colocasia esculenta* (L.) Schott.	Taro, Wild taro	Vegetable/tuber crop	Herb
Arecaceae	*Areca catechu* L.	Areca palm, Betel nut palm	Crop	Tree
	*Elaeis guineensis* Jacq.	Oil palm	Crop	Tree
Bromeliaceae	*Ananas comosus* (L.) Merr.	Pineapple	Fruit	Herb
Caricaceae	*Carica papaya* L.	Papaya	Fruit	Shrub
Combretaceae	*Terminalia catappa* L.	Indian almond tree	Crop	Tree
Convolvulaceae	*Ipomoea batatas* (L.) Lam.	Sweet potato	Tuber crop	Climber
Cucurbitaceae	*Coccinia indica* Wight & Arn.	Little gourd, Ivy gourd	Vegetable	Climber
	*Cucumis sativus* L.	Cucumber	Vegetable	Climber
	*Cucurbita moschata* (Duchesne ex Lam.)	Pumpkin	Vegetable	Climber
	*Momordiaca charantia* L.	Bitter gourd	Vegetable	Climber
	*Trichosanthes anguina* L.	Snake gourd	Vegetable	Climber
Dioscoriaceae	*Dioscorea alata* L.	Yam, Greater yam	Vegetable/tuber crop	Climber
Euphorbiaceae	*Manihot esculenta* Crantz.	Cassava	Tuber crop	Shrub
	*Ricinus communis* L.	Castor oil plant	Crop	Shrub
Fabaceae	*Acacia mangium* Willd.	Manjium	Timber crop	Tree
	*Arachis hypogea* L.	Groundnut, Peanut	Pulse	Herb
	*Cassia fistula* L.	Cassia	Ornamental	Tree
	*Gliricidia maculata* (Steud.)	Gliricidia, Quickstick	Weed	Tree
	*Vigna unguiculata* (L.) Walp.	Cowpea	Vegetable	Climber
Graminae	*Oryza sativa* L.	Rice	Cereal	Herb
	*Saccharum officinarum* L.	Sugarcane	Crop	Perennial Herb
Guttiferae	*Garcinia mangostana* L.	Mangostein	Fruit	Tree
Labiatae	*Ocimum sanctum* L.	Thulasi, Sacred basil	Medicinal	Shrub
Lauraceae	*Cinnamomum zeylanicum* Blume	Cinnamon	Spice	Tree
Malvaceae	*Abelmoschus esculentus* (L.) Moench	Bhindi, Okra, Ladies’ fingers, Gumbo	Vegetable	Herb
	*Hibiscus rosa*-*sinensis* L.	Shoe flower	Ornamental	Shrub
Marantaceae	*Maranta arundinacea* L.	Arrow root	Tuber crop	Herb
Moraceae	*Artocarpus heterophyllus* Lam.	Jack fruit tree	Fruit	Tree
	*Artocarpus hirsuitus* Lam.	Wild Jack	Fruit	Tree
	*Ficus religiosa* L.	Sacred fig, Peepal tree	Fruit	Tree
	*Morus alba*L.	Mulberry	Fruit	Shrub
Musaceae	*Musa paradisiaca* L.	Banana	Fruit	Herb
Myristicaceae	*Myristica fragrans* Houtt.	Nutmeg tree	Spice	Tree
Myrtaceae	*Syzygium aromaticum* (L.) Merr. & Perry	Clove tree	Spice	Tree
	*Psidium guajava* L.	Guava	Fruit	Tree
Orchidaceae	*Vanilla planifolia* Andr.	Vanila	Spice	Climber
Piperaceae	*Piper nigrum* L.	Pepper, Black pepper	Spice	Climber
Rubiaceae	*Coffea arabica* L.	Coffee, Arabian coffee	Crop	Shrub
	*Ixora javanica* (Blume) DC.	Asoka thechi, Jungle Geranium	Ornamental	Shrub
Rutaceae	*Citrus* × *aurantifolia* (Chistm. & Panz.) Swingle	Key lime	Fruit	Shrub
Simaroubaceae	*Ailanthus excelsa* Roxb.	Tree of Heaven	Timber crop	Tree
Solanaceae	*Capsicum annuum* L.	Chilli	Vegetable	Herb
	*Lycopersicum esculentum* Mill.	Tomato	Vegetable	Herb
	*Solanum melongena* L.	Brinjal, Eggplant, Aubergine	Vegetable	Shrub
Sterculiaceae	*Theobroma cacao* L.	Cocco, Cacao	Crop	Tree
Verbenaceae	*Tectona grandis* L.	Teak, Indian-oak	Timber crop	Tree
Zingiberaceae	*Cucurma longa* L.	Turmeric	Spice	Herb
	*Zingiber officinale* Rosc.	Ginger	Spice	Herb

* At least 50 plants of each species were observed. *Opisina arenosella* was found only on banana (*Musa paradisiaca* L.)

Leaf material from all the observed plant species was collected and used in subsequent laboratory evaluation of *O. arenosella* performance (see below). Similarly, lepidopteran larvae feeding on these plant species were collected for laboratory evaluations of parasitoid host range (see below). All subsequent laboratory work was conducted between 27 and 33 °C and 65 and 77% relative humidity.

### Screening of intercrop feeding by *O. arenosella* larvae

Leaves of all the intercrop species observed in the field were brought to the laboratory and screened for feeding by *O. arenosella* larvae. There were ten replicates for each intercrop plant species plus 10 replicates using coconut leaves. Leaves were selected haphazardly from plants of each species, using the same variety of plant within a species. In each replicate, a fresh leaf of uniform size within each species was presented to a single 3-week-old larva in a glass beaker (12 cm × 9 cm, Merck). Each leaf was checked for signs of feeding by *O. arenosella* larvae at 24-h intervals and replaced with a fresh leaf for five continuous days. When feeding was observed, the fed portions on the leaf were measured (using a Leica S8 APO Stereozoom trinocular microscope equipped with Leica Application Suite Version 4.2) to assess the area consumed by the larvae. The average daily rate of feeding by the larva was then calculated in terms of cm^2^ of leaf eaten per day. Data on feeding rates had non-constant variance across plant species (Bartlett’s test on residuals from Analysis of Variance [ANOVA] of feeding rate across plant species: *χ* ^2^ = 361.64, df = 24, *P* < 0.001) and were not normally distributed (Shapiro–Wilk test on residuals: *W* = 0.843, *P* < 0.001) so the effects of plant species on feeding rate were analysed using a nonparametric Kruskal–Wallis test (Siegel and Castellan 1988) in the GenStat statistical package (v.17.1, VSN International Ltd., Hemel Hempsted, UK).

### Performance of *O. arenosella* feeding on intercrop plants

**Fig. 3:**
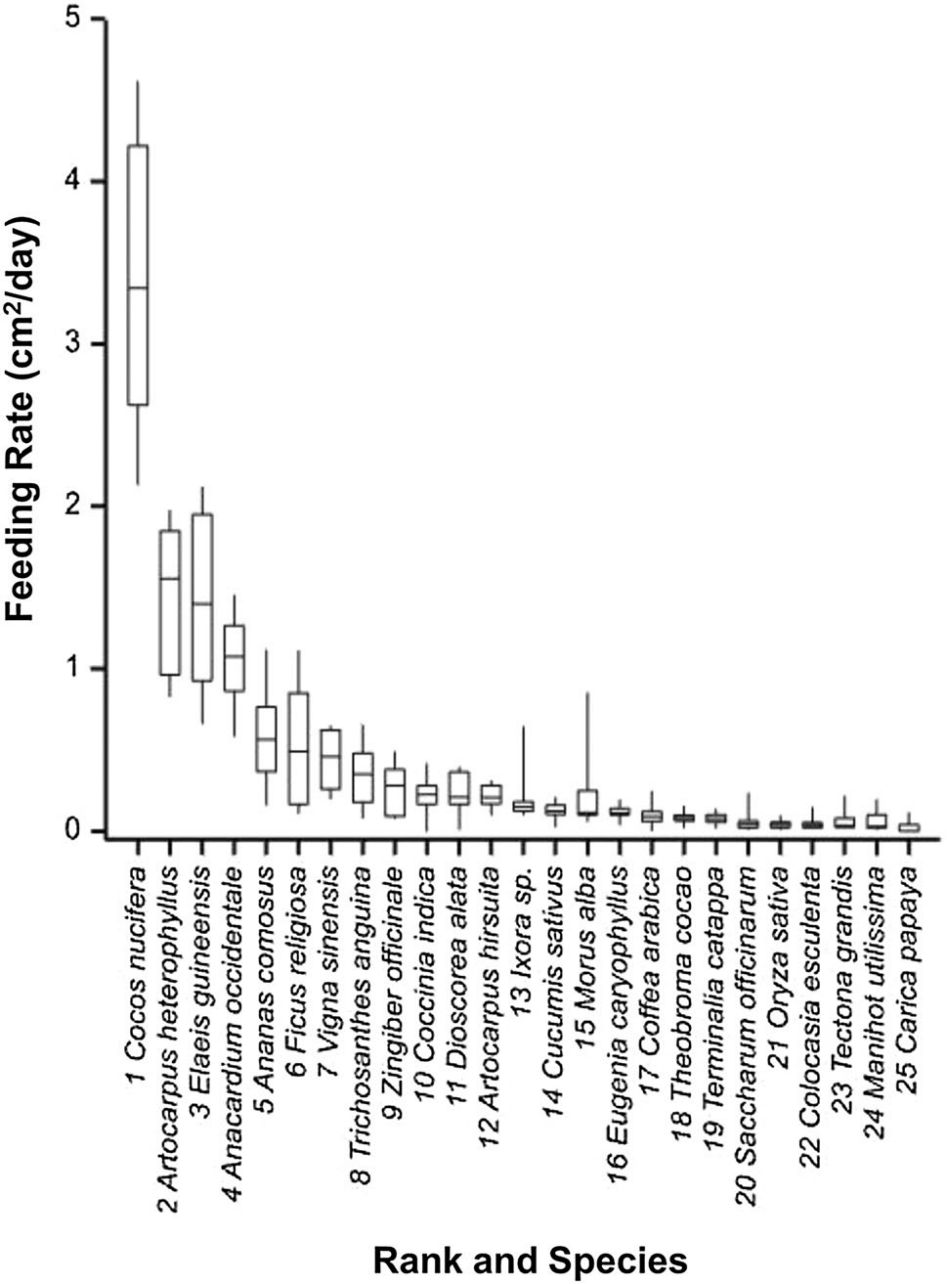
Feeding activity of *O. arenosella* larvae on coconut (*Cocos nucifera*) leaves and on leaves of 24 species of intercrops. Species are shown ranked according to median rate of feeding. Other species of intercrops tested were not fed on at all. *Bars* within boxes indicate medians, *ends of boxes* indicate upper and lower quartiles and *whiskers* indicate variability and skew

Studies on the survival and development of *O. arenosella* were conducted using the three host plants on which comparatively high feeding rates were observed (Fig. 3): *Artocarpus heterophyllus* (Jack fruit), *Elaeis guineensis* (Oil palm) and *Anacardium occidentale* (Cashew), plus coconut palm leaves (*C. nucifera*). In each replicate a freshly hatched first instar *O. arenosella* larva was transferred onto the leaf in a glass beaker, as above, and reared on them. Fresh leaves were provided every 48 h until pupation of *O. arenosella* larvae. Great care was taken during the transfer of early instar larvae onto fresh leaves; those which were injured or lethargic were excluded from the experiment, as were replicates in which the larva died. The larval period, pupal period and the longevity of the successfully developing adult female moths were recorded. For each plant species, there were ten replicates yielding adult moths. Data on the length of *O. arenosella* developmental stages and adult longevity had homogenous variance (Bartlett’s test on residuals from ANOVA of developmental time across plant species: Larval period, *χ* ^2^ = 5.13, df = 3, *P* = 0.163; Pupal period, *χ* ^2^ = 0.15, df = 3, *P* = 0.986; Longevity, *χ* ^2^ = 1.58, df = 3, *P* = 0.664) and were normally distributed (Shapiro–Wilk test: Larval period, *W* = 0.978, *P* = 0.632; Pupal period, *W* = 0.981, *P* = 0.714; Longevity, *W* = 0.974, *P* = 0.472). Thus, the effects of plant species on each of these measures were tested using one-way ANOVA in GenStat. Aggregation of factor levels was used to evaluate differences between treatments when overall results were significant (Crawley 1993). Once the minimal adequate (parsimonious) model was found, plots of the residuals against the fitted values, the standard normal distribution and plant species were used to check the assumptions of homogeneity of variance, normality and independence, respectively (Crawley 1993).

### Screening of intercrop herbivores as alternative hosts for parasitoids of *O. arenosella *

#### *Goniozus nephantidis*

Screening was carried out on 20 species of lepidopterans, belonging to six families, which were found on intercrop species (Table 2). Potential hosts were selected based on the size of the larvae being similar to those of *O. arenosella*. All larvae were collected from their respective intercrop host plants in the field and subsequently maintained in the laboratory on the leaves of these host plants until they developed to the size of a late-instar *O. arenosella* larva. The late-instar caterpillars were placed individually in glass tubes (10.5 × 2.5 cm, Borosil) containing a 5-day-old mated female *G. nephantidis*. The tubes were inspected daily under a stereomicroscope and any evidence of attack, oviposition and parasitoid development was recorded. Given a suitable host, *G. nephantidis* females normally sting and paralyze the larva on the day of exposure, typically lay eggs within 24 h and any eggs normally hatch around 1 day after being laid (S.K.S. & I.C.W.H. pers. obs.). To determine whether hosts were attacked, eggs were laid and if any hatched eggs developed as larvae, we observed each tube for 5 days. There were ten replicates for each of the 20 lepidopteran species.

**Table 2:** Lepidopterans found on intercrops as potential hosts for *Goniozus nephantidis* and *Brachymeria nosatoi*

Lepidopteran family	Species	Host plants	Parasitism^1^	
			*G. nephantidis*	*B. nosatoi*
Arctidae	*Amata passalis* Fab.	*Vigna unguiculata* (L.) Walp.	No	–
Cochlididae	*Contheyla rotunda* Hamp.	*Cocos nucifera* L.	No	No
	*Latoia lepida* Cram.	*Cocos nucifera* L.	No	No
Hesperidae	*Gangara thyrsis* Fab.	*Cocos nucifera* L.	No	No
	*Suastus gremius* Fb.	*Cocos nucifera* L.	No	–
Noctuidae	*Anadevidia peponis* (Fb.)	*Trichosanthes anguina* L.	No	No
	*Antoba olevaceae* Wlk.	*Solanum melongena* L.	No	No
	*Helicoverpa armigera* Hb.	*Vigna unguiculata* (L.) Walp., *Lycopersicum esculentum* Mill., *Trichosanthes anguina* L.	No	No
	*Spodoptera litura* (Fb.)	*Dioscorea alata* L., *Capsicum annuum* L., *Cucurbita moschata* (Duchesne ex Lam.), *Colocasia esculenta* (L.) Schott.	No	No
	*Turnaca acuta* W.	*Cocos nucifera* L.	No	No
Pieridae	*Catopsilia crocale* Cramer	*Cassia fistula* L.	No	No
Pyralidae	*Syllepte derogata* (Fb.)	*Abelmoschus esculentus* (L.) Moench	Yes (failed^2^)	Yes
	*Herculia nigrivita* Walker	*Cocos nucifera* L.	Yes (failed^2^)	Yes
	*Cnaphalocrocis medinalis* Guen.	*Oryza sativa* L.	No	–
	*Diaphania indica* Saund.	*Cucurbita moschata* (Duchesne ex Lam.), *Trichosanthes anguina* L., *Cucumis sativus* L.	No	No
	*Glyphodes glauculalis* Guen.	*Momordiaca charantia* L.	No	–
	*Leucinodes orbonalis* Guen.	*Trichosanthes anguina* L., *Solanum melongena* L.	No	–
	*Pilocrocis milvinalis*	*Cassia fistula* L.	No	No
	*Psara basalis* F.	*Amaranthus viridis* L.	No	–
	*Psara bipunctalis* Fb.	*Solanum melongena* L.	No	–

^1^Results follow presentation of late-instar larvae to *G. nephantidis* females and naked pupae to *B. nosatoi* females

^2^Hosts were stung and paralyzed and eggs were laid but offspring failed to develop

#### *Brachymeria nosatoi*

Screening for use as hosts by *B. nosatoi* Habu was carried out on 13 species of Lepidoptera, belonging to five families, which were found on intercrop species (Table 2). Potential hosts were selected on the basis of having similar size to *O. arenosella.* Potential hosts were collected in the larval stage from infested plants in the field, and reared on leaves of their respective host plants until they pupated. Since the naked (without silken cocoon) pupae of *O. arenosella* are readily accepted for oviposition by *B. nosatoi* females in the laboratory (S.K.S. pers. obs.), pupae of all species were also presented naked. Pupae were exposed individually to a 5-day-old mated female *B. nosatoi* for 4–5 h in a glass tube (10.5 × 2.5 cm, Borosil) and then kept in separate glass tubes until any parasitoids emerged and the development period was recorded. There were 10 replicates for each species of intercrop herbivores plus, for comparison, we also carried out 10 replicates using pupae of *O. arenosella* and 10 using pupae of the rice moth *Corcyra cephalonica* (Lepidoptera: Pyralidae), a factitious host of *Brachymeria* commonly used in mass-rearing facilities. Between host species differences in developmental period were tested for using ANOVA in GenStat. The residuals were homogenous and normally distributed (Bartlett’s test: *χ* ^2^ = 4.13, df = 4, *P* = 0.389; Shapiro–Wilk test: *W* = 0.975, *P* = 0.377).

### Construction of trophic webs for the coconut agro-ecosystem

A preliminary literature survey had revealed that many of the parasitoids of *O. arenosella* were also reported on pests of several other crops. Hence, an extensive literature survey was carried out to compile field records for coconut and all the intercrops grown in coconut plantations in the Indian subcontinent, all of their lepidopteran herbivores and the primary parasitoids of these herbivores. The sources from which information was collected were: Dharmaraju (1952, 1962), Balasundaram and Aiyadurai (1963), Nelliat et al. (1974), Varghese et al. (1978), Mohamed et al. (1982), Abdurahiman et al. (1983), Bavappa et al. (1986), Cock and Perera (1987), Paul (2007), Fatma and Pathak (2011), Sasidharan (2011), Sharma (2011), Muniappan et al. (2012), Yu et al. (2012), Sithanantham et al. (2013) and Noyes (2015). We supplemented these sources with records reported on the following web sites: The Plant List (2013), Indian Council of Agricultural Research—National Bureau of Agricultural Insect Resources (2013), Insects Catalog—Insecta.Pro (2007–2017), EPPO (2016).

These prior records were then used to compile composite connectance community webs (Memmott and Godfray 1994; Sunderland et al. 2005) of plant–herbivore and herbivore–parasitoid interactions within the coconut agro-ecosystem. Connectance, a proportional measure of community complexity, was calculated as the number of recorded herbivore–plant or parasitoid–herbivore interactions divided by the number of possible interspecific trophic interactions (Sunderland et al. 2005; Rocca and Greco 2015). We also quantified herbivore overlap and parasitoid overlap to indicate the degree to which sharing host plants and sharing natural enemies with intercrop herbivores might influence *O. arenosella* populations. Overlap was calculated as the number of pairs of species of plant, or herbivore, that shared at least one herbivore, or parasitoid, divided by the total possible number of such links (Sunderland et al. 2005). We also recorded the numbers of herbivore, or parasitoid, species that were shared between each linked pair of plants or herbivores, thus providing quantitative measures of overlap (van Veen et al. 2008).

## Results

### Field survey of intercrop plants

Across the four locations, a total of 54 species of intercrop plants, belonging to 34 families, were observed (Table 1). With the exception of banana in a heavily infested coconut plantation in Cochin during November 2010 to May 2011, there were no observations of *O. arenosella* on any intercrops in any location either during the infestation or non-infestation periods. However, larvae of 20 other species of Lepidoptera were collected from the intercrop plants (see below).

### Screening of intercrop feeding by *O. arenosella* larvae

*Opisina arenosella* larvae fed on the leaves of 24 species of intercrops, but not on the other 30 species screened. Feeding rates differed significantly among the intercrop species which were fed on (Kruskal–Wallis test: *H* = 178.8, df = 23, *P* < 0.001, Fig. 3). The most fed on intercrop species were *Artocarpus heterophyllus* (Jack fruit), *Elaeis guineensis* (Oil palm) and *Anacardium occidentale* (Cashew), where feeding rates were approximately 30–45% of those observed on *C. nucifera* (Coconut) leaves. Leaves of all other intercrop species were fed on at rates lower than 20% of the rate of feeding on coconut; nonetheless, the leaves of around 10 further species also found to be acceptable to *O. arenosella* larvae (Fig. 3). However, it was also observed that feeding on *Ananas comosus* (Pineapple) and *Terminalia catappa* (Indian almond) led to premature pupation of some larvae.

### Performance of *O. arenosella* feeding on intercrop plants

The *O. arenosella* larvae constructed its characteristic gallery on jack fruit, cashew and oil palm exactly in the same manner as in coconut (Fig. 1). The adult moths that emerged appeared to be morphologically and physiologically normal. The larval period differed significantly across all four plant species presented and was shortest when on coconut (Table 3). Pupal periods were also shortest on coconut but did not differ among larvae fed on the three intercrops (Table 3). The longevity of adult females was unaffected by the species of plant on which larvae had fed (Table 3).

**Table 3:** The mean developmental periods and adult longevity of *O. arenosella* reared on different plant species

Plant species	Larval period (days ± SE)	Pupal period (days ± SE)	Adult female longevity (days ± SE)
*Artocarpus heterophyllus* (Jackfruit)	51.33 ± 0.68^a^	9.33 ± 0.30^a^	4.33 ± 0.26
*Anacardium occidentale* (Cashew)	47.14 ± 0.46^b^	9.00 ± 0.33^a^	4.71 ± 0.40
*Elaeis guineensis* (Oil palm)	42.60 ± 0.91^c^	8.60 ± 0.31^a^	4.00 ± 0.37
*Cocos nucifera* (Coconut)	36.20 ± 0.96^d^	7.00 ± 0.30^b^	4.72 ± 0.367
*F*_(3,36)_	68.679	10.948	0.941
*P*	<0.001	<0.001	0.431

For larval and pupal data, the superscript letters within columns indicate whether responses to each plant species were similar (assessed by aggregation of factor levels)

Statistical results are from ANOVA

### Screening of intercrop herbivores as alternative hosts for parasitoids of *O. arenosella *

#### Goniozus nephantidis

Only three of the intercrop herbivore species presented were attacked by *G. nephantidis* females, all were members of the family Pyralidae (Table 2). While the larvae of *H. nigrivita* and *Syllepte* (=*Sylepta*) *derogata* were stung, paralyzed and had eggs laid on them, and the eggs hatched to larvae, the parasitoids did not complete development as the host either decayed or became desiccated within 2 days.

#### *Brachymeria nosatoi*

Of the 13 species of intercrop herbivores screened, *B. nosatoi* successfully parasitized the pupae of the Pyralid species *H. nigrivita, S. derogata* and *Psara basalis* (Table 2). The naked pupae of these three species were readily accepted, and the oviposition behaviour was the same as described by Mohamed et al. (1983) on *O. arenosella* pupae. Pupae of the factitious host *C. cephalonica* were also attacked similarly. The developmental period of *B. nosatoi* varied significantly across host species: development was most rapid on *O. arenosella,* slowest on *Corcyra cephalonica* and intermediate (and not significantly different) across the three intercrop herbivore species (Table 4).

**Table 4:** The developmental period of *B. nosatoi* on different species of host pupae

Lepidopteran species	Developmental period (days ± SE)
*Opisina arenosella*	12.5 ± 0.269^a^
*Corcyra cephalonica*	15.6 ± 0.400^b^
*Herculia nigrivita*	14.0 ± 0.211^c^
*Syllepte derogata*	13.7 ± 0.335^c^
*Psara basalis*	14.0 ± 0.365^c^
*F*_(4,45)_	11.710
*P*	<0.001

Superscript letters indicate whether responses to each host species were similar (assessed by aggregation of factor levels)

Statistical results are from ANOVA

### Trophic webs

**Fig. 4:**
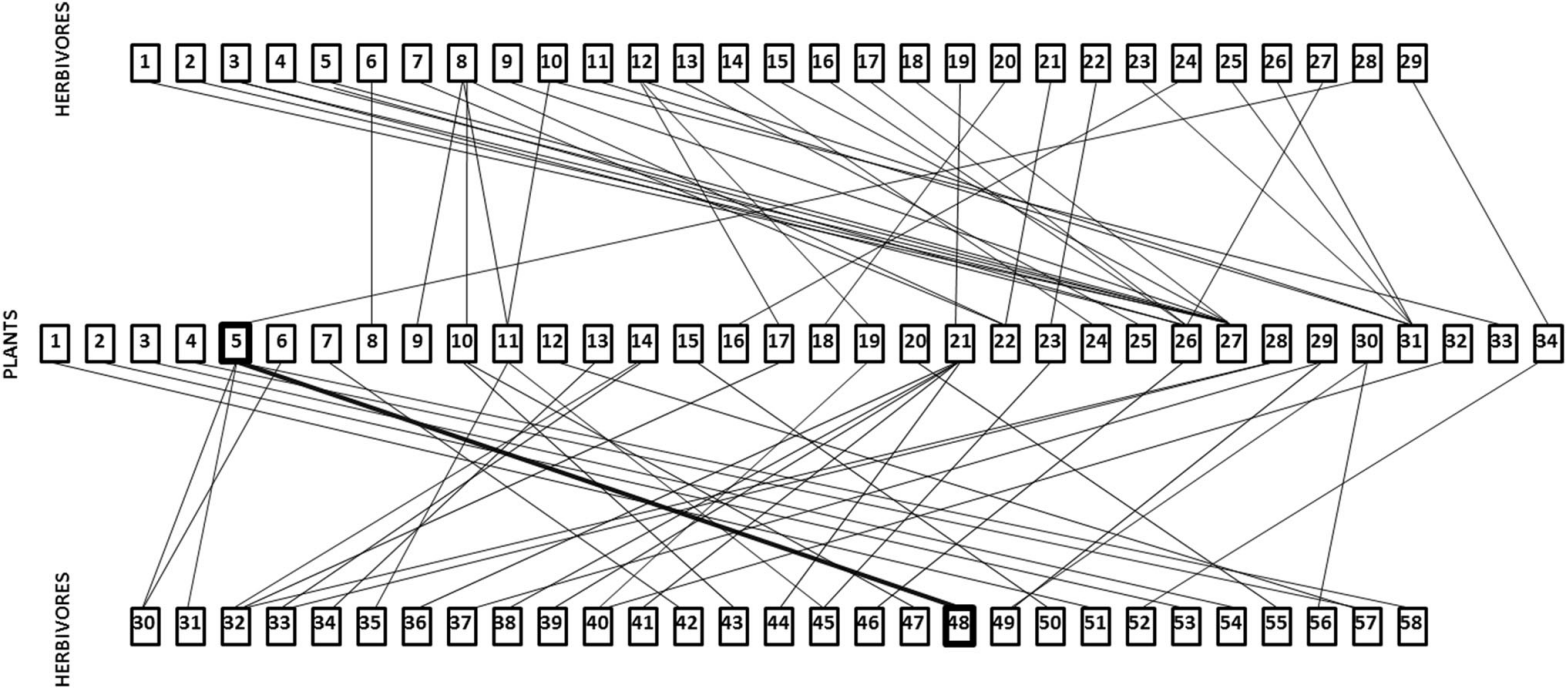
Trophic interactions between plants and herbivores in coconut plantations. Composite connectance web summarizing herbivory within the coconut plantation community. Coconut (*C. nucifera*) is highlighted with a thick box, as is its major pest *O. arenosella*. All herbivores listed belong to the Lepidoptera. Plants. Amaranthaceae: 1*. Amaranthus viridis* L. Anacardiaceae: 2. *Anacardium occidentale* L., 3. *Mangifera indica* L. Araceae: 4. *Amorphophalus paeonifolius* (Dennst.) Nicolson. Arecaceae: 5. *Cocos nucifera* L., 6. *Elaeis guineensis* Jacq. Bromeliaceae: 7. *Ananas comosus* (L.). Caricaceae: 8. *Carica papaya* L. Cucurbitaceae: 9. *Coccinia indica* Wight & Arn., 10. *Cucumis sativus* L., 11. *Trichosanthes anguina* L. Dioscoriaceae: 12. *Dioscorea alata* L. Euphorbiaceae: 13. *Manihot esculenta* Crantz., 14. *Ricinus communis* L. Fabaceae: 15. *Acacia mangium* Willd., 16. *Arachis hypogea* L., 17. *Phaseolus* spp., 18. *Tamarindus indica* L. 19. *Vigna unguiculata* (L.) Walp. Lamiaceae: 20. *Ocimum tenuiiflorum* L. Malvaceae: 21. *Abelmoschus esculentus* (L.) Moench. Moraceae: 22. *Artocarpus heterophyllus* Lam., 23. *Morus alba* L. Moringaceae: 24. *Moringa oleifera* Lam. Musaceae: 25. *Musa paradisiaca* L. Poaceae: 26. *Oryza sativa* L., 27. *Saccharum officinarum* L. Punicaceae: 28. *Punica granatum* L. Rubiaceae: 29. *Ixora javanica* (Blume) DC. Rutaceae: 30. *Citrus* × *aurantifolia* (Chistm. & Panz.) Swingle. Solanaceae: 31. *Capsicum annuum* L., 32. *Lycopersicum eseulentum* Mill. 33. *Solanum melongena* L.Verbenaceae: 34. *Tectona grandis* L. Herbivores. Crambidae: 1. *Chilo infuscatellus* Snellen, 2. *Chilo partellus* (Swinhoe), 3. *Chilo polychrysus* (Meyrick), 4. *Chilo sacchariphagus indicus* (Kapur), 5. *Chilo suppressalis* Walker, 6. *Conogethes punctiferalis* (Guenée), 7. *Diaphani acaesalis* (Walker), 8. *Diaphania indica* (Saunders), 9. *Diatraea saccharalis* (Fabricius), 10. *Leucinodes orbonalis* Guenée, 11. *Maruca* sp., 12. *Maruca vitrata* (Fabricius), 13. *Nacoleia octasema* (Meyrick), 14. *Noorda moringae* Tams, 15. *Scirpophaga excerptalis* (Walker), 16. *Scirpophaga incertulas* (Walker), 17. *Scirpophaga innotata* (Walker), 18. *Scirpophaga nivella* (Fabricius), 19. *Syllepte derogate* Fabricius. Erebidae: 20. *Dasychira* sp., 21. *Perina nuda* (Fabricius), 22. *Spilosoma obliqua* (Walker), 23. *Utetheisa pulchella* (Linnaeus). Gelechiidae: 24. *Aproaerema modicella* Deventer, 25. *Pectinophora gossypiella* (Saunders), 26. *Phthorimaea operculella* (Zeller). Hesperiidae: 27. *Pelopidas mathias* (Fabricius), 28. *Suastus gremius* (Fabricius). Hyblaeidae: 29. *Hyblaea puera* (Cramer). Limacodidae: 30. *Contheyla rotunda* Hampson, 31. *Parasa lepida* Cramer. Noctuidae: 32. *Achaea janata* (Linnaeus), 33. *Achaea* sp., 34. *Agrotis ipsilon* (Hufnagel), 35. *Anadevidia peponis* (Fabricius), 36. *Anomis flava* (Fabricius), 37. *Chrysodeixis includens* (Walker), 38. *Earias insulana* Boisduval, 39. *Earias vittela* (Fabricius), 40. *Helicoverpa armigera* (Hübner), 41. *Helicoverpa zea* (Boddie), 42. *Mythimna* sp., 43. *Peridroma saucia* (Hübner), 44. *Spodoptera exigua* Hübner, 45. *Spodoptera litura* (Fabricius), 46. *Spodoptera mauritia* (Boisduval), 47. *Trichoplusia ni* (Hübner). Oecophoridae: 48. *Opisina arenosella* Walker. Papilionidae: 49. *Papilio polytes*. Pieridae: 50. *Eurema* sp. Plutellidae: 51. *Plutella xylostella* (Linnaeus). Pyralidae: 52. *Eutectona machaeralis* (Walker), 53. *Lamida moncusalis* Walker, 54. *Orthaga exvinacea* (Hampson). Saturniidae: 55. *Antheraea mylitta* (Drury). Sphingidae: 56. *Agrius convolvuli* (Linnaeus), 57. *Hippotion celerio* (Linnaeus). Zygaenidae: 58. *Artona catoxantha* Hampson

The literature survey provided records for coconut plus 33 species of intercrops, 58 species of lepidopteran herbivores and 29 species primary parasitoids. Coconut was reported to be fed on by five herbivore species, its major pest *O. arenosella* plus *Suastus gremius*, *Contheyla rotunda*, *Parasa lepida* and *Artona catoxantha* (Fig. 4), while the number of herbivores feeding on intercrop species ranged between 1 and 8 (mean = 2.09). The connectance of the plant–herbivore trophic web was 0.038 (Fig. 4). Overall, the proportion of herbivore overlap between the plant species was low, 0.034, with around half of the plant species (14/34) not sharing any herbivores at all, although two pairs of plant species were linked by more than one herbivore (Table 5). Coconut shared a herbivore, *C. rotunda*, with only one intercrop species, oil palm, *E. guineensis* (Table 5).

**Table 5:**
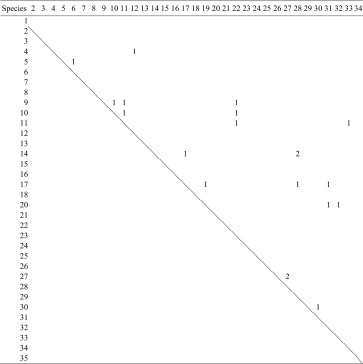
Herbivore overlap among coconut and the 33 species of intercrops

The numbers of herbivore species shared between each possible pair of plants are shown. The proportion of pairs sharing at least one herbivore was 0.034. Plant species identities are as given in Fig. 4

**Fig. 5:**
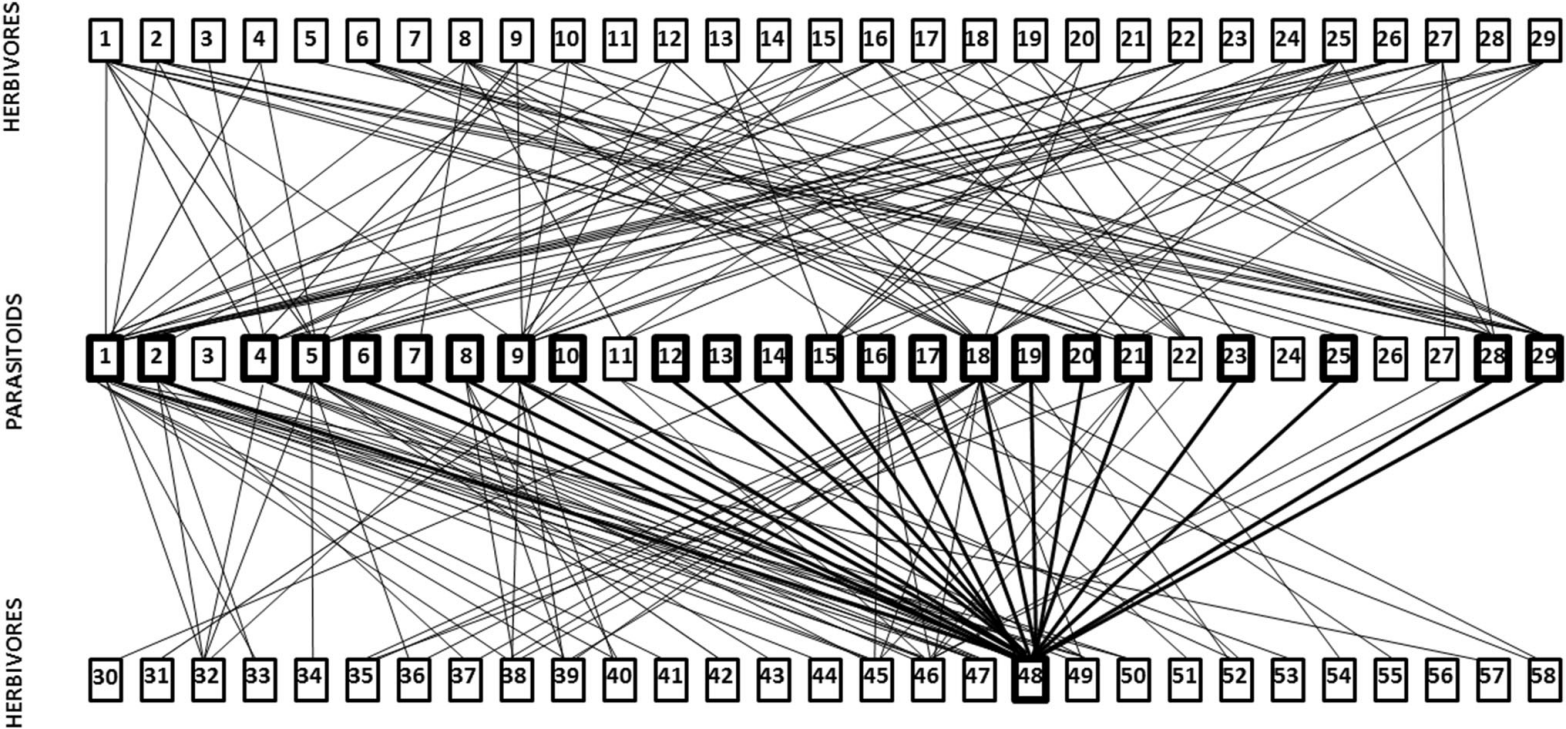
Trophic interactions between herbivores and parasitoids in coconut plantations Composite connectance web summarizing the Herbivore—Parasitoid complex within the coconut plantation community. The coconut caterpillar *O. arenosella* and its parasitoids are highlighted with thick boxes and bold lines. Herbivores: All belong to Lepidoptera, as given in Fig. 4. Egg Parasitoids. Trichogrammatidae: 1. *Trichogramma chilonis* Ishii, 2. *Trichogramma evanescens* Westwood, 3. *Trichogramma exiguum* Pinto & Platner, 4. *Trichogramma japonicum* Ashmead, 5. *Trichogramma minutum* Riley. Larval Parasitoids. Bethylidae: 6. *Goniozus nephantidis* (Musebeck). Braconidae: 7. *Apanteles taragamae* Viereck, 8. *Bracon brevicornis* Wesmael, 9. *Bracon hebetor* Say, 10. *Fornicia ceylonica* Wilkinson. Eulophidae: 11. *Elasmus brevicornis* Gahan, 12. *Elasmus nephantidis* Rohwer. Pupal Parasitoids. Braconidae: 13. *Meteoridea hutsoni* Nixon. Chalcididae: 14. *Antrocephalus hakonensis* Ashmead, 15. *Brachymeria euploeae* Westwood, 16. *Brachymeria excarinata* Gahan, 17. *Brachymeria hime atteviae* Joseph, Narendran & Joy, 18. *Brachymeria lasus* Walker, 19. *Brachymeria nephantidis* Gahan, 20. *Brachymeria nosatoi* Habu. Eulophidae: 21. *Tetrastichus howardi* Olliff, 22. *Tetrastichus schoenobii,* 23. *Trichospilus pupivorus* Ferrière. Ichneumonidae: 24. *Eriborus ricini* Rao & Kurian, 25. *Eriborus trochanteratus* Morely, 26. *Trathala flavoorbitalis* Cameron, 27. *Xanthopimpla flavolineata* Cameron, 28. *Xanthopimpla punctata* Fabricius, 29. *Xanthopimpla stemmator* Thunberg

The connectance of the herbivore–parasitoid trophic web (Fig. 5) was 0.112. *Opisina arenosella* was attacked by four species of egg parasitoids, six larval parasitoids and 13 pupal parasitoids (Fig. 5). Overall, the proportion of parasitoid overlap between the herbivore species was 0.472 (Table 6). Pairs of herbivore species that were linked by shared parasitism, shared an average of 1.611 species of parasitoids. *O. arenosella* shared a mean of 2.317 parasitoid species with each other herbivore species to which it was linked by parasitism (Table 6).

**Table 6:**
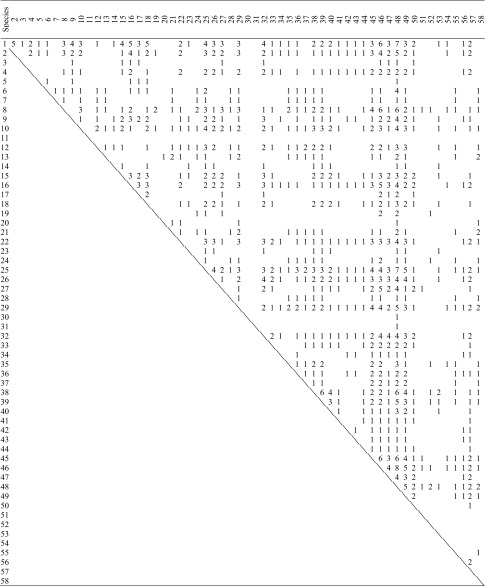
Parasitoid overlap among herbivores

The numbers of parasitoid species shared between each possible pair of herbivores species are shown. The proportion of pairs sharing at least one parasitoid is 0.472. Herbivore species identities are as given in Fig. 4

We further calculated overall overlap separately for egg, larval and pupal parasitism: egg parasitoid overlap was 0.303, larval parasitoid overlap was 0.086 and pupal parasitoid overlap was 0.203. In terms of the numbers of parasitoids shared by pairs herbivores linked by parasitism, the mean number of shared egg parasitoid species was 1.381, mean shared number of larval parasitoids was 1.077 and mean shared pupal parasitoids was 1.235.

## Discussion

*Opisina arenosella* can feed on several plant species that are planted as intercrops with coconut, for instance forming characteristic galleries of frass and silk when feeding on the leaves of jack fruit, cashew and oil palm. While banana was not fed on in the laboratory, the occurrence of *O. arenosella* on banana in a severely infested coconut plantation at Cochin, where jack fruit or cashew or oil palm were not available (S.K.S. pers. obs.), further illustrates that intercrop species may be utilized during severe pest outbreaks. However, others may be entirely unsuitable due to chemical or physical deterrents (e.g. Schuman et al. 2016; Zhang et al. 2017), which could account for the premature pupation of *O. arenosella* when fed on pineapple and Indian almond leaves. In all cases, the performance of *O. arenosella* on intercrops was lower than when feeding on coconut and it is seldom found feeding on intercrops in the field. As such, the role intercrops may play in the direct maintenance of *O. arenosella* populations does not seem likely to be substantial. Further, *O. arenosella* is unlikely to be in direct competition for resources with other lepidopterans originating from intercrops (which could lead to competitive exclusion, Reitz and Trumble 2002; van Veen et al. 2006a, 2008) as these herbivores do not typically feed on coconut.

Similarly, intercrops are unlikely to influence directly *G. nephantidis*, the major parasitoid of *O. arenosella* larvae, as it does not develop to maturity on the intercrop herbivores we screened. *Goniozus nephantidis* can, however, be reared on other factitious hosts belonging to several lepidopteran families (including the Pyralid *C. cephalonica*) and is widely used in the augmentative biological control of *O. arenosella* (Dharmaraju 1952; Nirula 1956; George et al. 1977; Remadevi et al. 1978, 1996; Mohamed et al. 1982; Sathiamma et al. 1987; Mohan and Shameer 2003; Rajan et al. 2009). The paralysis of, and oviposition on, *H. nigrivita* and *S. derogata* indicates that *G. nephantidis* may contribute to suppression of these intercrop herbivores by killing larvae, even if subsequent parasitism is unsuccessful. In contrast, *B. nosatoi*, a major parasitoid of *O. arenosella* pupae, that is also mass reared and released in the augmentative biocontrol (Joy and Joseph 1972, 1973; Sathiamma et al. 1987), is able to develop on the pupae of several species of intercrop herbivores: *S. derogata*, *H. nigrivita* and *P. basalis* were successfully parasitized in the laboratory. *Brachymeria nosatoi* readily accepted naked pupae of all the five pests in the laboratory and development of *B. nosatoi* on *S. derogata*, *H. nigrivita* (*contra* Mohamed et al. 1983) and development on *P. basalis* was significantly faster than on the commonly used factitious host *C. cephalonica*. Hence, the pupae of these three pest species can be utilized for the mass multiplication of *B. nosatoi* in the laboratory. The acceptance of naked pupae of *C. cephalonica* by *B. nosatoi* is advantageous for the laboratory multiplication of this parasitoid given the fact that *C. cephalonica* can be easily reared in the laboratory. Our empirical results suggest that intercrops can harbour populations of *B. nosatoi* and thus also that *O. arenosella* and intercrop herbivores may interact indirectly via shared *B. nosatoi* parasitism.

Trophic webs constructed from literature evidence similarly suggest that intercrop plants share relatively few lepidopteran herbivores among themselves or with coconut but that there is a considerable degree of natural enemy sharing among intercrop herbivores and *O. arenosella*. In terms of the number of species involved, the sharing of egg and pupal parasitoids is more prevalent in the agro-ecosystem than is the sharing of larval parasitoids. We suggest that this may reflect a greater degree of co-evolutionary intimacy (e.g. involving host immune responses and parasitoid countermeasures) between larval parasitoids and their hosts than between egg- or pupal parasitoids and their hosts (see also van Veen et al. 2008 for an analogous argument). *Goniozus nephantidis* appears to be monophagous within the agro-ecosystem (despite having a number of factitious laboratory hosts, see above) and *Elasmus nephantidis,* which is recommended for field release against *O. arenosella* (Sathiamma et al. 1987), and the larval-pupal parasitoid *Meteoridea hutsoni*, which is commonly found in infested palms, are also not reported from any intercrop herbivores. However, *Apanteles taragamae,* a solitary endoparasitoid of second and third instar *O. arenosella* larvae, is commonly found in infested coconut plantations and also attacks the larvae of *Diaphania indica* which infests *Coccinia grandis* and *Cucumis sativus* L., vegetable crops which are often intercrops of coconut. Egg parasitoids appear to be considerably less host specific. For instance, *Trichogramma chilonis*, *T. minutum* and *T. evanescens,* which are parasitoids of *O. arenosella eggs* (Mohamed et al. 1982), also attack 26, 20 and 11 species of intercrop herbivores, respectively. Similarly, a number of pupal parasitoids of *O. arenosella* can be reared on other lepidopteran host species (Kabeerathumma and Nair 1971; Nadarajan and Jayaraj 1975; Pillai and Nair 1987; Baitha et al. 2003). For example, five species of *Brachymeria*, which are pupal parasitoids of *O. arenosella*, can develop on many of the intercrop herbivores (Joy et al.^1978^; Mohamed et al. 1982, 1983; Streito and Nibouche 1997); in particular, *B. nosatoi* was reported from *C. punctiferalis* (infesting *C. papaya*) and *Pectinophora gossypiella* (infesting *Capsicum annuum*). Biocontrol practitioners have observed that *B. nosatoi* is often the first parasitoid species to reach new *O. arenosella* outbreaks (Pers. Comm. from staff of the Coconut Research Institute, Lunuwila, Sri Lanka) and this characteristic may be promoted by its ability to develop on these intercrop herbivores.

Our estimates of trophic web connectance between plants and herbivores (0.038) and between herbivores and parasitoids (0.112) are lower than values reported from a cropping system recently introduced into the new world and containing a small number of species (0.39, among 4 host and 6 parasitoid species, Rocca and Greco 2015). This may be due to a false working assumption that all plant, Lepidopteran and parasitoid species all belong to the same ecological community (Poulin 2010), essentially delineated by planation boundaries. Our connectance estimates are, however, similar to some values reported for natural communities of herbivores and parasitoids (0.06–0.10, among 45 hosts and 31 parasitoids, Maunsell et al. 2015). One interpretation could be that parasitoid trophic webs in coconut plantations are, in fact, naturalistic due to this being a long-established agro-ecosystem. Cross-study comparisons between connectance estimates must, however, be made with caution as values are sensitive to sampling limitations, which will increase mechanistically in larger webs and may explain the lower estimates among webs containing more species (Blüthgen 2010; Poulin 2010).

Although our empirical and literature-based evaluations do not assess the population densities of the insect species in the agro-ecosystem or quantify the strengths of interactions in the trophic web (Memmott and Godfray 1994; Valladares and Salvo 1999; Sunderland et al. 2005; Maunsell et al. 2015; Rocca and Greco 2015), current evidence suggests that indirect ecological interactions, such as apparent competition (Holt and Lawton 1993; Müller and Godfray 1997; van Veen et al. 2006a,b, 2008; Jaworski et al. 2015), via shared parasitoids could influence populations of *O. arenosella* when intercrops are present. Apparent competition may ultimately exclude all but one herbivore species from a community and the dominant (remaining) species may be that which supports the highest density of parasitoids (Holt and Lawton 1993; van Veen et al. 2006a). In terms of number of species (the connectance trophic web, Fig. 5, provides no measures of densities), *O. arenosella* is a host of 23 of the 29 parasitoids in the agro-ecosystem, substantially more than any of the other lepidopterans recorded, which suggests that *O. arenosella* may be affected, but perhaps not excluded, by apparent competition, during periods of infestation. Further, the presence of apparent competition in agro-ecosystems does not necessarily reduce pest damage (Jaworski et al. 2015). The potential for intercrop herbivores to sustain parasitoids of *O. arenosella* during non-infestation periods is, however, clear and this is likely to promote pest suppression by decoupling parasitoid populations from the constraining seasonality of *O. arenosella* availability (Settle et al. 1996; Holt and Hochberg 2001; Clementine et al. 2005; Feng et al. 2017). In other agro-ecosystems, specialist insect herbivores have been shown to exhibit lower population densities in diverse habitats containing host and non-host plants compared with simple habitats containing host plants only (Kareiva 1983; Risch et al. 1983; Stanton 1983; Andow 1988). As parasitoids specialized on each of the developmental stages (egg, early instar larvae, late-instar larvae, pupal) of *O. arenosella* are shared with intercrop herbivores, detrimental interspecific competition between parasitoids (Hardy and Blackburn 1991; Denoth et al. 2002) may be reduced and pest populations may be additively suppressed (Hassell 1978; Kindlman and Ruzicka 1992).

Attaining an understanding of the composition and dynamics of ecological and agro-ecological communities is extremely challenging, as the forms and strengths of species interactions are varied and complex (e.g. Watt 1965; Paine 1992; Holt and Lawton 1993; Wilson et al. 1996; Valladares and Salvo 1999; Holt and Hochberg 2001; van Veen et al. 2006a; Poulin 2010; Allesina and Tang 2012; Jaworski et al. 2015; Levine et al. 2017). The information we provide and synthesize here can at present serve only as a tentative guide towards more detailed understanding of the dynamics of the coconut agro-ecosystem. The literature records we compile may be biased due to greater attention having paid to some species than to others. The construction of semi-quantitative or quantitative trophic webs directly from field observations would constitute a desirable extension of this work, as would the inclusion of non-lepidopteran herbivores, predators, pathogens, and hyper-parasitoids into these webs (Memmott and Godfray 1994; Valladares and Salvo 1999; van Veen et al. 2006a, 2008; Maunsell et al. 2015; Rocca and Greco 2015). An understanding of why given parasitoid species attack some intercrop herbivores, but not others could be gained by metabolomic analysis (Snart et al. 2015) to identify biochemical differences between herbivore species, or tri-trophic effects of the host plants fed upon (Bukovinszky et al. 2008; Schuman et al. 2016), that might prevent extreme polyphagy and thus the wider sharing of natural enemies. Further, direct observations that parasitoids developing on intercrop herbivores subsequently attack *O. arenosella* would provide key evidence for whether coconut and its intercrops form single or segregated habitats (Feng et al. 2017).

Since the practice of intercropping/mixed cropping in large-scale coconut plantations and homestead gardens is very common, the recommendation of specific crops or plants to be grown along with coconut in the context of beneficial plant–herbivore–parasitoid associations is likely to aid the conservation biocontrol of *O. arenosella*. Indeed, from our own experience in Kerala (near Alappuzha) it seems that *O. arenosella* infestations are less severe in areas with intercrops than in those without (pers. obs. S.K.S & C.M.), and we note that these observations accord with the conclusions of recent meta-analyses across a wide range of agro-ecosystems (Letourneau et al. 2011; Iverson et al. 2014). We recommend the following intercrops to be grown in coconut plantations because of their support for major parasitoids of *O. arenosella* (parasitoids are in parenthesis): *Cucumis sativus (T. minutum, A. taragammae, B. lasus, X. punctata), Morus alba (T. minutum, B. lasus), Oryza sativa (T. minutum, B. lasus, X. punctata), Saccharum officinarum (T. minutum, B. hebetor, X. punctata), Tectona grandis (T. minutum, B. lasus), Abelmoschus esculentus (T. minutum, B. hebetor, B. lasus, X. punctata), Capsicum annuum (T. minutum, B. hebetor, B. lasus, B. nosatoi, X. punctata), Citrus aurantifolia (T. minutum, B. hebetor), Ricinus communis (T. minutum, B. hebetor), Coccinia grandis (A. taragammae, B. lasus, X. punctata), Trichosanthes anguina (B. hebetor, B. lasus), Carica papaya (B. lasus, B. nosatoi)* and *Solanum melongena (B. lasus, X. punctata)*. Not every plant species will be suited to every coconut plantation, for example due to variation in soil types and water profiles, but growing at least some of these plants is expected to be beneficial. Further work will be required to establish whether and how different combinations of intercrops might affect the population biology of *O. arenosella*.

## Conclusions

Our empirical evaluations and the construction of trophic webs from prior literature both suggest that the presence of intercrops will not greatly affect *O. arenosella* populations, either by providing substantial alternative food sources for this pest species or by promoting direct herbivore–herbivore competition between the pest and other lepidopterans. In contrast, the high degree of parasitoid overlap between the herbivores present indicates that indirect competitive interactions, such as apparent competition, are likely play an important role in the coconut agro-ecosystem. These patterns accord with conclusions drawn by a number of prior studies of natural and semi-natural communities of phytophagous insects (van Veen et al. 2006a, b). The dynamics of multi-species host–parasitoid communities in nature and in agro-ecosystems are expected to be complex and inferences concerning the effects of shared parasitoids are thus constrained to be tentative. Current evidence nonetheless seems sufficient to allow us to recommend a number of intercrop species that are most likely to promote the suppression of *O. arenosella* via indirect ecological interactions, although we recognize that it may not be practicable to grow intercrops in all areas where coconut is cultivated and that any effects of different combinations of intercrops remain unexplored. Intercrops are most likely to exert an influence by maintaining populations of parasitoids during seasons in which *O. arenosella* at stages suitable for parasitism are scarce. While intercrops may not enhance natural enemy action in every agro-ecosystem, we consider it likely that our inferences will apply to other cropping systems in addition to coconut.

## Author contribution

Shameer K.S. gathered the empirical and trophic web data, analysed the data and wrote the manuscript. Nasser M. instigated the research and commented on the manuscript. Chandrika Mohan discussed the data and commented on the manuscript. I.C.W. Hardy analysed the data and wrote the manuscript.
